# Supplementary figures and images for: Genetic variation of Nigerian cattle inferred from maternal and paternal genetic markers
Source: PeerJ. 2021 Mar 5;9:e10607. doi: 10.7717/peerj.10607 (PMC7938780; doi:10.7717/peerj.10607)

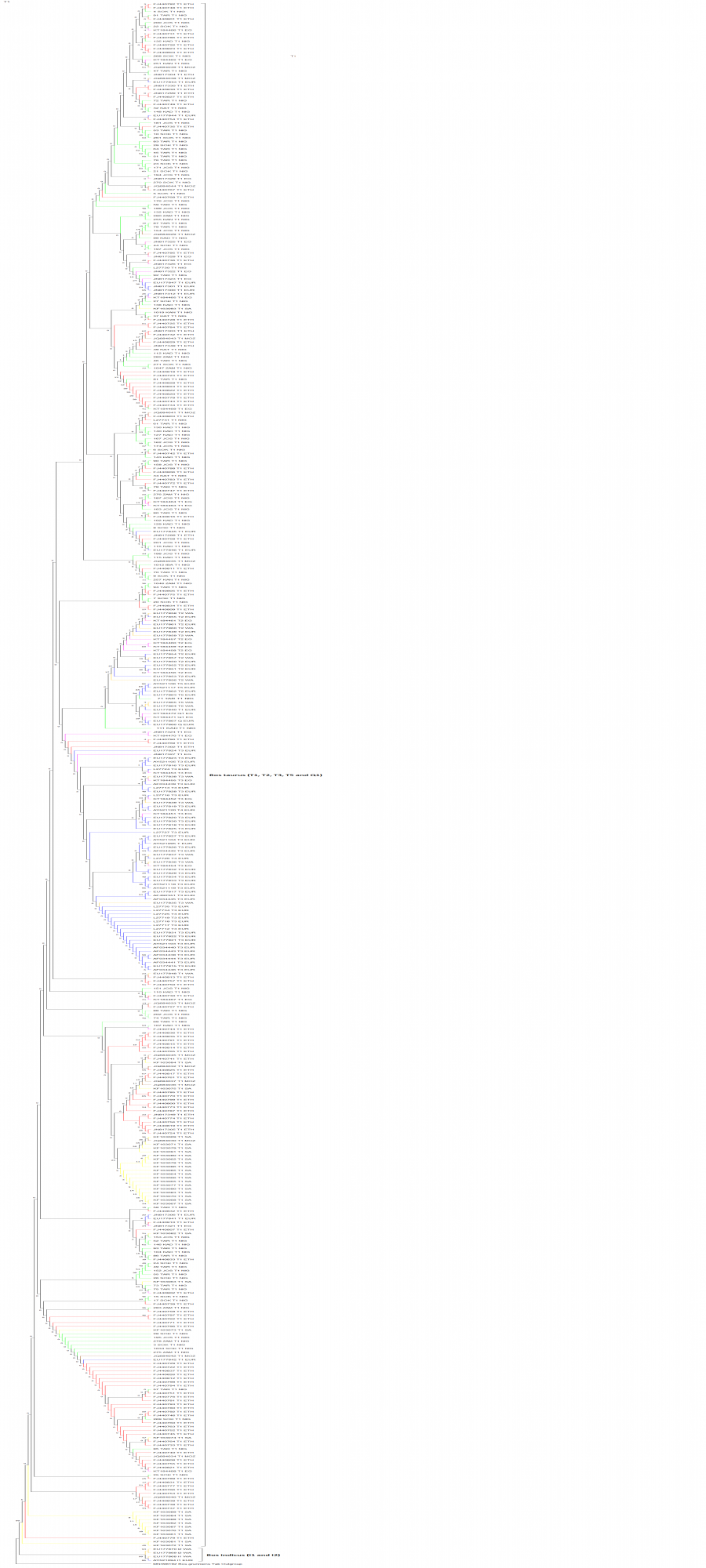

Supplement: Supplemental Information 1 — Colours of the branch lengths represent samples from different geographical locations: Green-Nigeria, Pink-Egypt, Brown-Mozambique, Red-Ethiopia, Yellow-South Africa, Blue-Europe, Orange-West Asia. [file peerj-09-10607-s001.png]

**a**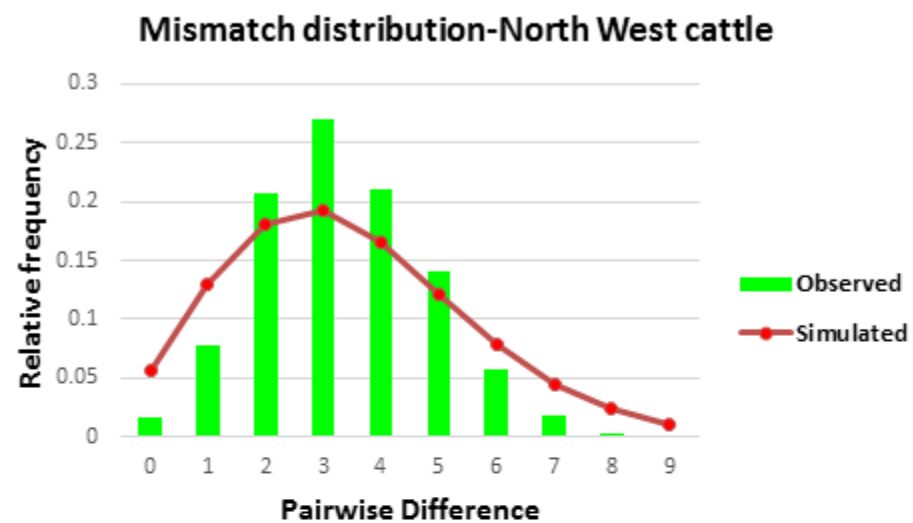**b**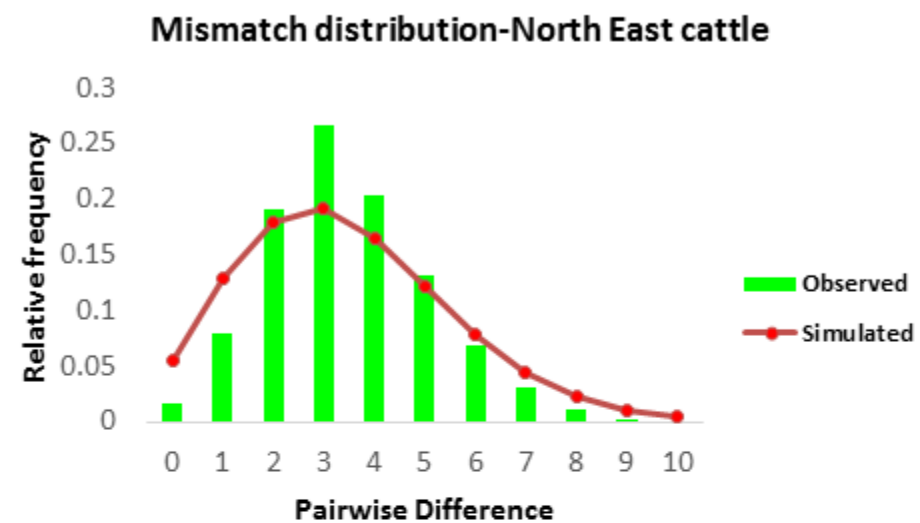**c**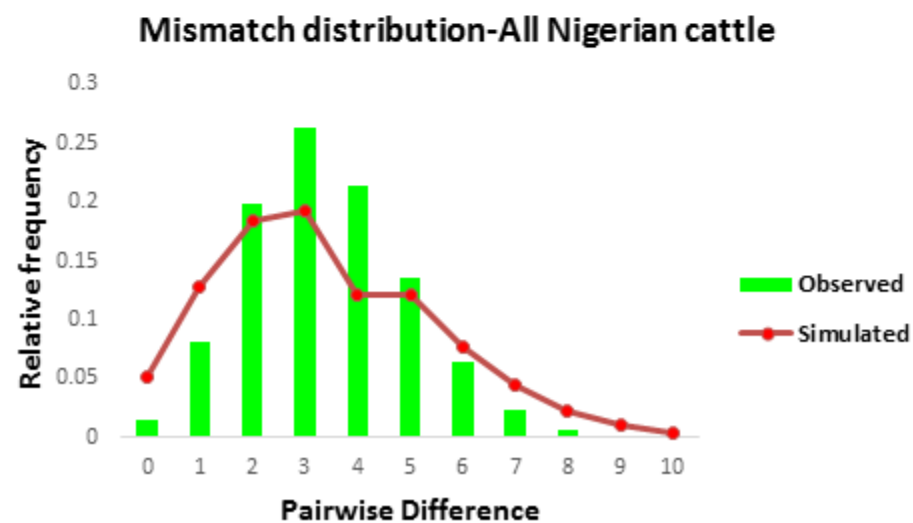

Supplement: Supplemental Information 2 — Mismatch distribution patterns for Nigerian cattle samples from (A) North West region (B) North East region and (C) the overall Nigerian cattle as a whole. [file peerj-09-10607-s002.pdf]

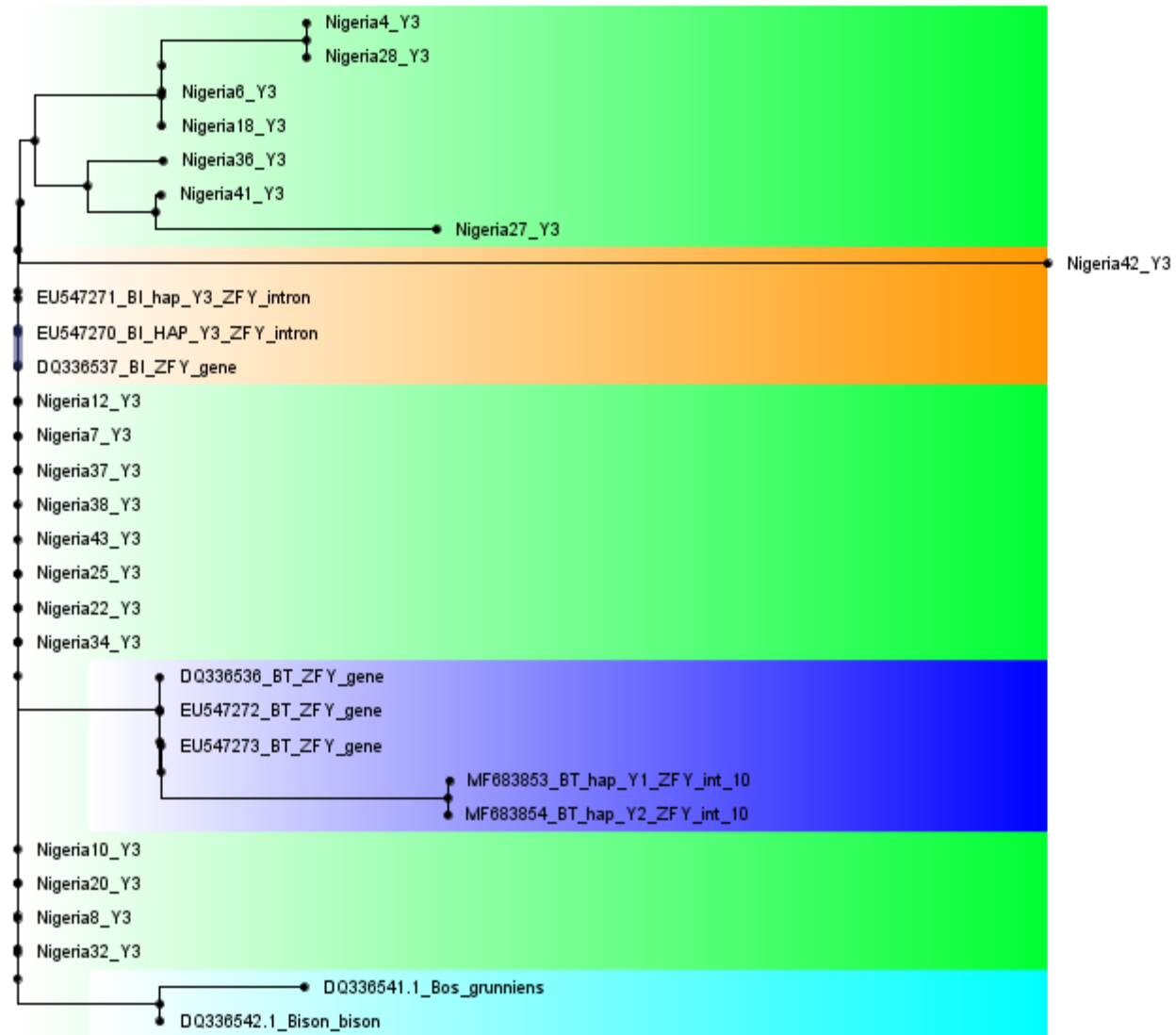

0.003

Supplement: Supplemental Information 4 — Clade colours represent samples fromdifferent geographical locations: Green-Nigeria, Blue-Europe, Orange-South Asia, Cyan- Outgroups. [file peerj-09-10607-s004.pdf]
