# Supplementary material for: Genetic variation of Nigerian cattle inferred from maternal and paternal genetic markers
Source: PeerJ. 2021 Mar 5;9:e10607. doi: 10.7717/peerj.10607 (PMC7938780; doi:10.7717/peerj.10607)
Supplement: Supplemental Information 3 — Source of the data for the haplogroups was retrieved from Perez-Pardal et al. (2018), Ginja, Telo da Gama & Penedo (2009) and that conducted in this study. [file peerj-09-10607-s003.pdf]

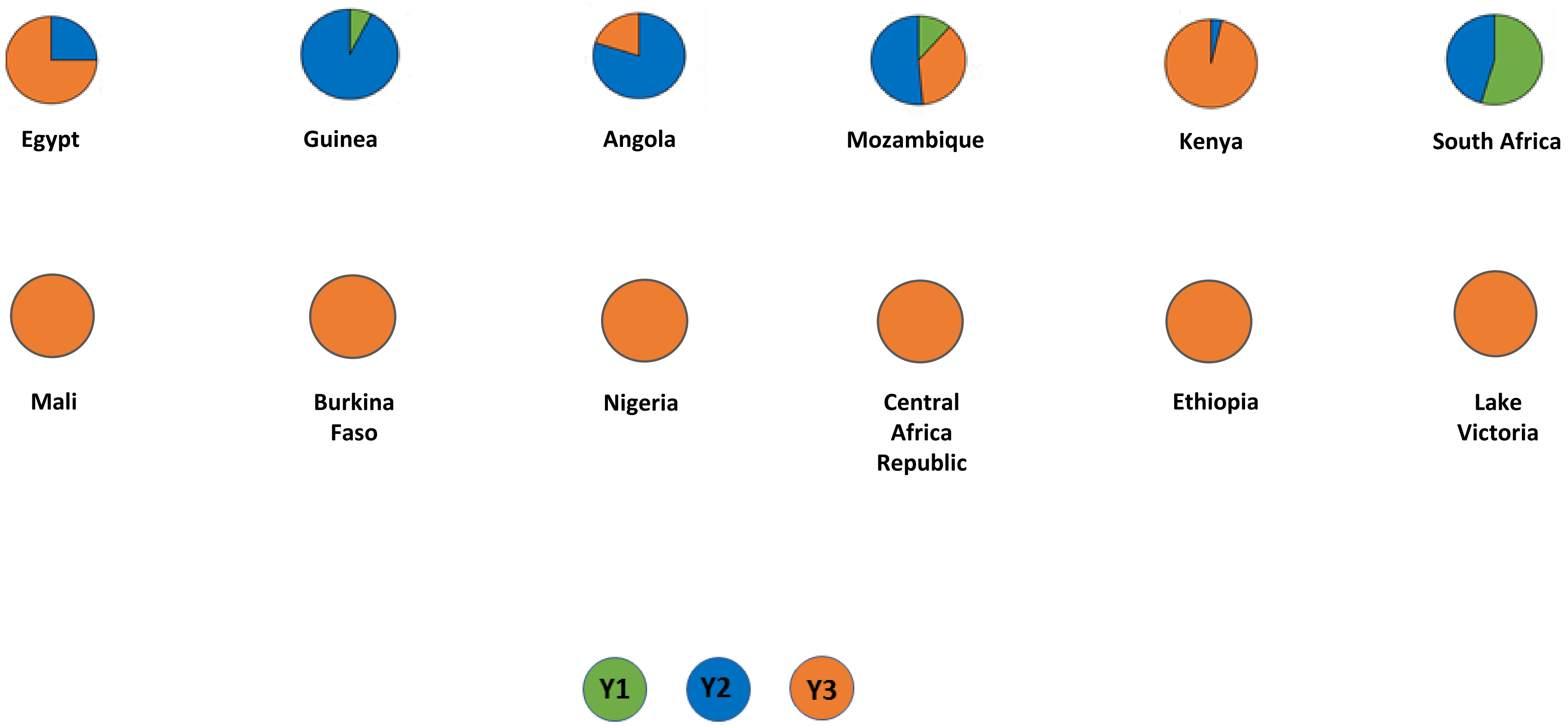
Egypt
Guinea
Angola
Mozambique
Kenya
South Africa
Mali
Burkina Faso
Nigeria
Central Africa Republic
Ethiopia
Lake Victoria
Y1
Y2
Y3
