# Supplementary material for: Genetic variation of Nigerian cattle inferred from maternal and paternal genetic markers
Source: PeerJ. 2021 Mar 5;9:e10607. doi: 10.7717/peerj.10607 (PMC7938780; doi:10.7717/peerj.10607)
Supplement: Supplemental Information 9 [file peerj-09-10607-s009.docx]

**Table S5. Variants information present in Nigerian cattle based on mtDNA**

| **S/NO** | **Sample Name** | **Variants** |
| --- | --- | --- |
| 1 | 202_JOS_NIG | 16015,16068,16255 16015,16050,16068,16113,16255 |
| 2 | 22_SOK_NIG | 68T,16050,16113,16255 |
| 3 | 23_SOK_NIG | 16127,16255,16050,16113,16127,1625 |
| 4 | 28_SOK_NIG | 16255,16022,16050,16113,1625 |
| 5 | 24_SOK_NIG | 112C,114,16022,16050,16113,16133,16255 |
| 6 | 25_SOK_NIG | 16085,16255,16050,16085,16113,1625 |
| 7 | 26_SOK_NIG | 123G,16133,16255,16301,123G,16022,16050,16113,16133,16255,1630 |
| 8 | 27_SOK_NIG | 8,16095,16249,16255,16260 |
| 9 | 102_KAD_NIG | 106,16050,16074,16113,16139,16255 |
| 10 | 10_SOK_NIG | 16050,16051,16113,16197,16255 |
| 11 | 1012_IBA_NIG | 16050,16113,16131,16195,16255 |
| 12 | 1019_KAN_NIG | 16050,16113,16247 |
| 13 | 1046_ZAM_NIG | 16050,16113,16231 |
| 14 | 129_KAD_NIG | 106,16050,16113,16255 |
| 15 | 130_KAD_NIG | 16050,16057,16113,16255,16301 |
| 16 | 132_KAD_NIG | 15965,16050,16094,16113,16255 |
| 17 | 135_KAD_NIG | 16050,16113,16255 |
| 18 | 138_KAD_NIG | 16050,16113,16255,16260,16301 |
| 19 | 140_KAD_NIG | 16050,16057,16113,16255,16301 |
| 20 | 143_KAD_NIG | 106,16050,16113,16255,16301 |
| 21 | 15_SOK_NIG | 16022,16050,16084,16113,16138,16255 |
| 22 | 154_JOS_NIG | 16050,16113,16121,16255 |
| 23 | 17_SOK_NIG | 16022,16050,16084,16113,16138,16255 |
| 24 | 171_JOS_NIG | 16050,16113,16127,16255 |
| 25 | 195_JOS_NIG | 16022,16050,16113,16255 |
| 26 | 21_SOK_NIG | 16050,16113,16127,16255 |
| 27 | 269_SOK_NIG | 16022,16050,16113,16121,16255 |
| 28 | 276_ZAM_NIG | 106,16050,16113,16126,16197,16255 |
| 29 | 278_ZAM_NIG | 16022,16050,16113,16255 |
| 30 | 280_ZAM_NIG | 15965,16050,16113,16255 |
| 31 | 29_SOK_NIG | 16050,16113,16232,16255 |
| 32 | 3_SOK_NIG | 16022,16050,16113,16255 |
| 33 | 32_KAT_NIG | 16050,16113,16138,16255 |
| 34 | 34_KAT_NIG | 106,16022,16050,16113,16255 |
| 35 | 37_KAT_NIG | 16050,16113 |
| 36 | 39_KAT_NIG | 15921,16050,16113,16197,16247,16255 |
| 37 | 4_SOK_NIG | 16050,16113,16255 |
| 38 | 44_SOK_NIG | 16050,16113,16135,16255 |
| 39 | 5_SOK_NIG | 16050,16113,16255 |
| 40 | 52_TAR_NIG | 16050,16057,16085,16113,16248,16255 |
| 41 | 53_TAR_NIG | 16050,16051,16113,16255 |
| 42 | 55_TAR_NIG | 16022,16050,16113,16133,16255 |
| 43 | 57_TAR_NIG | 15958,16022,16050,16113,16139,16255 |
| 44 | 58_TAR_NIG | 16022,16050,16113,16248,16255 |
| 45 | 59_TAR_NIG | 16050,16108,16113,16119,16255 |
| 46 | 6_SOK_NIG | 16050,16113,16255,16301 |
| 47 | 61_TAR_NIG | 16050,16057,16113,16255 |
| 48 | 64_TAR_NIG | 16050,16113,16232,16255 |
| 49 | 69_TAR_NIG | 16050,16056,16113,16255 |
| 50 | 7_SOK_NIG | 16050,16057,16113,16231,16255 |
| 51 | 70_TAR_NIG | 16050,16113,16195,16255 |
| 52 | 71_TAR_NIG | 15921,16113,16197,16255 |
| 53 | 72_TAR_NIG | 16050,16113,16255 |
| 54 | 8_SOK_NIG | 106,16050,16113,16255 |
| 55 | 87_TAR_NIG | 16050,16113,16121,16255 |
| 56 | 88_TAR_NIG | 15953,16050,16068,16113,16139,16255 |
| 57 | 9_SOK_NIG | 16050,16113,16195,16255 |
| 58 | 90_TAR_NIG | 106,107,16022,16050,16113,16255 |
| 59 | 91_TAR_NIG | 15939,16050,16113,16255 |
| 60 | 92_TAR_NIG | 16050,16108,16113,16122,16131,16196,16255 |
| 61 | 93_TAR_NIG | 16050,16113,16248,16255 |
| 62 | 94_TAR_NIG | 16050,16113,16231 |
| 63 | 99_KAD_NIG | 16050,16074,16113,16121,16135,16247,16255 |
| 64 | 104_KAD_NIG | 16050,16113,16248,16255 |
| 65 | 146_KAD_NIG | 16050,16057,16085,16113,16248,16255 |
| 66 | 148_KAD_NIG | 16050,16109,16113,16138,16255 |
| 67 | 152_JOS_NIG | 16022,16050,16113,16133,16255 |
| 68 | 153_JOS_NIG | 16050,16085,16113,16248,16255,16260,16264 |
| 69 | 158_JOS_NIG | 106,16022,16050,16112,16113,16250,16255 |
| 70 | 162_JOS_NIG | 100,123G,16050,16113,16255 |
| 71 | 163_JOS_NIG | 106,113,123G,15965,16050,16113,16195,16255 |
| 72 | 167_JOS_NIG | 123G,16050,16057,16113,16255 |
| 73 | 174_JOS_NIG | 123G,15959,16050,16113,16255 |
| 74 | 176_JOS_NIG | 16050,16113,16255,16302 |
| 75 | 181_JOS_NIG | 16050,16113,16255 |
| 76 | 187_JOS_NIG | 106,113,16050,16113,16126,16255 |
| 77 | 1034_SOK_NIG | 16022,16050,16113,16255 |
| 78 | 275_ZAM_NIG | 16022,16050,16113,16255 |
| 79 | 199_JOS_NIG | 16050,16108,16113,16255 |
| 80 | 261_SOK_NIG | 16050,16051,16113,16197,16255 |
| 81 | 200_JOS_NIG | 16050,16113,16255 |
| 82 | 268_SOK_NIG | 16050,16113,16255 |
| 83 | 270_SOK_NIG | 16050,16113,16255 |
| 84 | 194_JOS_NIG | 16050,16113,16255 |
| 85 | 283_ZAM_NIG | 16016,16022,16050,16058,16113,16255 |
| 86 | 198_JOS_NIG | 16050,16053,16113,16195,16255 |
| 87 | 197_JOS_NIG | 8,16050,16113,16131,16135,16255 |
| 88 | 282_ZAM_NIG | 16050,16113,16247,16255 |
| 89 | 255_KAN_NIG | 15965,16050,16113,16135,16141,16255 |
| 90 | 20_SOK_NIG | 16050,16057,16113,16231,16255 |
| 91 | 271_SOK_NIG | 106,16050,16113,16130,16247,16255 |
| 92 | 257_KAN_NIG | 16050,16113,16195,16255 |
| 93 | 251_KAN_NIG | 16050,16058,16113,16143,16255 |
| 94 | 201_JOS_NIG | 106,16050,16067,16113,16255 |
| 95 | 45_TAR_NIG | 16050,16113,16232,16255 |
| 96 | 46_TAR_NIG | 16050,16113,16139,16247,16255 |
| 97 | 47_TAR_NIG | 15930,16050,16113,16255 |
| 98 | 49_TAR_NIG | 16022,16050,16113,16133,16255 |
| 99 | 51_TAR_NIG | 16050,16113,16232,16255 |
| 100 | 1047_ZAM_NIG | 106,16050,16113,16130,16247,16255 |
| 101 | 73_TAR_NIG | 16022,16050,16113,16255,16301 |
| 102 | 74_TAR_NIG | 15915,16050,16068,16113,16255 |
| 103 | 75_TAR_NIG | 16022,16050,16113,16255,16301 |
| 104 | 76_TAR_NIG | 16050,16113,16255 |
| 105 | 78_TAR_NIG | 106,16050,16053,16084,16113,16255,16301,16302 |
| 106 | 79_TAR_NIG | 16050,16113,16121,16255 |
| 107 | 80_TAR_NIG | 106,16016,16050,16113,16255 |
| 108 | 81_TAR_NIG | 16022,16050,16113,16247,16255 |
| 109 | 83_TAR_NIG | 16050,16113,16255 |
| 110 | 85_TAR_NIG | 16022,16050,16113,16126,16255 |
| 111 | 86_TAR_NIG | 16050,16113,16248,16255 |
| 112 | 110_KAD_NIG | 16022,16050,16057,16074,16113,16141,16255 |
| 113 | 111_KAD_NIG | 16113,16204,16255 |
| 114 | 112_KAD_NIG | 16050,16113,16247,16255 |
| 115 | 115_KAD_NIG | 16050,16053,16113,16255 |
| 116 | 116_KAD_NIG | 106,15894,16050,16113,16255 |
| 117 | 127_KAD_NIG | 16050,16057,16113,16255,16301 |
| 118 | 151_JOS_NIG | 15949,16050,16074,16113,16131,16255 |
| 119 | 107_KAD_NIG | 15830,16022,16050,16113,16255 |
