# Supplementary material for: Genetic variation of Nigerian cattle inferred from maternal and paternal genetic markers
Source: PeerJ. 2021 Mar 5;9:e10607. doi: 10.7717/peerj.10607 (PMC7938780; doi:10.7717/peerj.10607)
Supplement: Supplemental Information 10 [file peerj-09-10607-s010.docx]

**Table S6.** Assessment of the geographical structure within and among cattle populations using AMOVA based on mtDNA D-loop sequences.

| Hierarchical clusters | Variance components | % of variation | F | *P*-value |
| --- | --- | --- | --- | --- |
| 1. Nigerian population (overall) | Among population | 0.88 | 0.00877 | 0.18964 |
|  | Within population | 99.12 |  |  |
|  |  |  |  |  |
|  |  |  |  |  |
| 2. Nigeria vs. ^a^ South Africa | Among groups | 34.32 | 0.34324 | 0.33040 |
|  | Among  populations  within  groups | -1.60 | -0.02436 | 0.88074 |
|  | Within populations | 67.28 | 0.32724 | 0.00000 |
|  |  |  |  |  |
| 3. Nigeria vs. ^b^ Mozambique | Among groups | 8.15 | -0.08146 | 0.26002 |
|  | Among  populations  within  groups | -1.61 | -0.01751 | 0.47996 |
|  | Within populations | 93.46 | 0.06538 | 0.00196 |
|  |  |  |  |  |
| 4. Nigeria vs. ^c^ Egypt | Among groups | 1.24 | 0.01244 | 0.64614 |
|  | Among  populations  within  groups | 15.70 | 0.15893 | 0.01173 |
|  | Within populations | 83.06 | 0.16940 | 0.00000 |
|  |  |  |  |  |
| 5. Nigeria vs. ^d^ Ethiopia | Among groups | 6.85 | 0.06853 | 0.09189 |
|  | Among  populations  within  groups | -0.11 | -0.00113 | 0.47312 |
|  | Within populations | 93.25 | 0.06748 | 0.00000 |
|  |  |  |  |  |
| 6. Nigeria vs. ^e^ Europe | Among groups | 26.52 | 0.26523 | 0.33272 |
|  | Among  populations  within  groups | 5.42 | 0.07375 | 0.00004 |
|  | Within populations | 68.06 | 0.31941 | 0.00000 |
|  |  |  |  |  |
| 7. Nigeria vs. ^f^ West Asia | Among groups | 30.39 | 0.30389 | 0.33553 |
|  | Among  populations  within  groups | 7.33 | 0.10533 | 0.55841 |
|  | Within populations | 62.28 | 0.37722 | 0.00000 |
|  |  |  |  |  |

Note:  ^a^ South Africa: Nguni cattle populations; ^b^ Mozambique: Landim, Angone and Tete cattle breeds; ^c^ Egypt: Domiaty and Menofi cattle populations; ^d^ Ethiopia: Adaw, Ambo, Arsi, Boran, Danakil, Fogera, Horro, Ogaden, Raya-Azebo and Sheko cattle breeds; ^e^ Europe: Cattle from Italy, Greece, Portugal, Spain, and Republic of Ireland; ^f^ West Asia: Iranian and Iraqi cattle breeds
