# Supplementary material for: Genetic variation of Nigerian cattle inferred from maternal and paternal genetic markers
Source: PeerJ. 2021 Mar 5;9:e10607. doi: 10.7717/peerj.10607 (PMC7938780; doi:10.7717/peerj.10607)
Supplement: Supplemental Information 11 [file peerj-09-10607-s011.docx]

**Table S7.** The population pairwise differentiation measured by genetic distance F*_ST_*

(i) The F*_ST_* pairwise estimates for population differentiation among eight Nigerian cattle sub-populations estimated at number of permutations 1,000

| Population | Taraba | Plateau | Zamfara | Katsina | Kaduna | Kano | Sokoto | Oyo |
| --- | --- | --- | --- | --- | --- | --- | --- | --- |
| Taraba | 0 |  |  |  |  |  |  |  |
| Plateau | 0.007 | 0 |  |  |  |  |  |  |
| Zamfara | -0.005 | 0.007 | 0 |  |  |  |  |  |
| Katsina | -0.037 | -0.029 | -0.124 | 0 |  |  |  |  |
| Kaduna | 0.019 | **0.022** | 0.029 | -0.018 | 0 |  |  |  |
| Kano | 0.036 | 0.005 | -0.026 | -0.071 | 0.025 | 0 |  |  |
| Sokoto | -0.007 | 0.008 | -0.006 | -0.038 | **0.029** | 0.051 | 0 |  |
| Oyo | 0.064 | 0.010 | 0.117 | 0.084 | 0.055 | 0.028 | 0.098 | 0 |

(ii) The F*_ST_* pairwise estimates for population differentiation between Nigerian cattle and other populations from Africa, Europe and West Asia estimated at 1,000 replications

| Population | Nigeria | Europe | Egypt | Ethiopia | Mozambique | South Africa | West Asia |
| --- | --- | --- | --- | --- | --- | --- | --- |
| Nigeria | 0 |  |  |  |  |  |  |
| Europe | **0.30724** | 0 |  |  |  |  |  |
| Egypt | **0.13325** | **0.08178** | 0 |  |  |  |  |
| Ethiopia | **0.06795** | **0.34775** | **0.18172** | 0 |  |  |  |
| Mozambique | **0.04159** | **0.28502** | **0.11382** | -0.00548 | 0 |  |  |
| South Africa | **0.33054** | **0.42889** | **0.3193** | **0.23989*** | **0.22678*** | 0 |  |
| West Asia | **0.36406** | **0.11701** | **0.09995** | **0.38724*** | **0.19852*** | **0.36459** | 0 |

Note: The significant F*_ST_ P*-values at significance level 0.05 are given in bold
