# Supplementary material for: Genetic variation of Nigerian cattle inferred from maternal and paternal genetic markers
Source: PeerJ. 2021 Mar 5;9:e10607. doi: 10.7717/peerj.10607 (PMC7938780; doi:10.7717/peerj.10607)
Supplement: Supplemental Information 12 [file peerj-09-10607-s012.docx]

**Table S8.** The goodness of fit and neutrality tests for demographic expansion and population growth estimated from mtDNA D-loop in 119 Nigerian cattle

| **Population** | **Neutrality test** | | **Mismatch distribution** | | | **Goodness of fit test** | |
| --- | --- | --- | --- | --- | --- | --- | --- |
|  | **Tajima's D (p)** | **Fu's FS (p)** | **Tau** | **Theta 0** | **Theta 1** | **SSD (p)** | **HRI (p)** |
| North West | -2.143 (0.005) | -26.243 (0.000) | 3.000 | 0.550 | 3414.978 | 0.008 (0.001) | 0.042 (0.002) |
| North East | -2.366 (0.002) | -26.160 (0.000) | 3.000 | 0.550 | 3414.978 | 0.005 (0.013) | 0.037 (0.004) |
| West | 0.000 (1.000) | -0.341 (0.191) | 3.000 | 0.550 | 3414.978 | NA | NA |
| All | -2.3145 (0.000) | -26.203 0.000) | 3.000 | 0.550 | 3414.978 | 0.006 (0.001) | 0.0381 (0.0381) |

Note: SSD = Sum of Squared deviation, HRI = Harpending's Raggedness index, p = P-value
