# Supplementary material for: Genetic variation of Nigerian cattle inferred from maternal and paternal genetic markers
Source: PeerJ. 2021 Mar 5;9:e10607. doi: 10.7717/peerj.10607 (PMC7938780; doi:10.7717/peerj.10607)
Supplement: Supplemental Information 13 [file peerj-09-10607-s013.docx]

**Table S9.** Genetic diversity of cattle in Africa based on Y-chromosomal microsatellite markers and ZFY intron 10 gene

| **Population** | **N** | **HD (SD)** | |
| --- | --- | --- | --- |
| 1. Nigeria ^p^ | 6 | 0.800(0.213) |  |
| 2. Egypt ^q^ |  |  |  |
| (a) Baladi | 2 | 0.500 |  |
| (b) Damiata | 2 | 0.000 |  |
| 3. Mozambique ^q^ |  |  |  |
| (a) Angone | 8 | 0.842 |  |
| (b) Landim | 18 | 0.852 |  |
| (c) Tete | 2 | 0.500 |  |
| 4. Ethiopia ^p^ | 9 | 0.750(0.169) |  |
| 5. South Africa ^q^ | 11 | 0.496 |  |
| 6. Kenya ^p^ | 29 | 0.357(0.029) |  |
| 7. Burkina Faso ^p^ |  |  |  |
| (a) Lobi | 15 | 0.429(0.056) |  |
| (b) Zebu_Peul | 35 | 0.235(0.007) |  |
| 8. Central African Republic ^p^ | 6 | 0.800(0.213) |  |
| 9. Mali ^p^ | 28 | 0.444(0.046) |  |
| 10. Angola ^q^ | 5 | 0.498 |  |
| 11. Lake Victoria ^q^ | 13 | 0.320 |  |
| 12. Guinea ^q^ |  |  |  |
| (a) Bafata | 8 | 0.719 |  |
| (b) Gabu | 11 | 0.810 |  |

|  | **H | 20 |  |  |
| --- | --- | --- | --- | --- |
|  | **PS | 10 |  |  |
|  | **HD (SD) | 1.000 (0.016) | |  |
|  | **Df (SD) | 1.679 (1.027) | |  |

Note: N = sample size, PS = the number of polymorphic sites, HD = haplotype diversity, and SD = standard deviations, Df = the mean number of nucleotide differences and ** = Genetic diversity of Nigerian cattle. ^p^ Data retrieved from *Perez-Pardal et al. (2018)*. ^q^ Data retrieved from *Ginja et al. (2019)*.
